# Factors affecting demand for individual health insurance in Malaysia

**DOI:** 10.1186/1471-2458-12-S2-A10

**Published:** 2012-11-27

**Authors:** Arpah Abu Bakar, Angappan Regupathi, Syed Mohamed Aljunid, Mohd Azahadi Omar

**Affiliations:** 1 College of Business, Universiti Utara Malaysia, 06010 UUM Sintok, Malaysia; 2 United Nations University-International Institute for Global Health, Universiti Kebangsaan Malaysia Medical Center, Jalan Yaacob Latiff, 56000 Kuala Lumpur, Malaysia; 3 Institute for Public Health, Ministry of Health Malaysia, Jalan Bangsar, 50590 Kuala Lumpur, Malaysia

## Background

Private health insurance is one of the sources of funds for financing health care apart from direct taxes, public insurance and out of pocket payments. Regardless of the choice between a social and private insurance, health insurance ownership has been linked with lower OOP. Thus, health insurance as a type of health care financing, no doubt, will be one of the financing mechanisms in the health care financing model. As such, understanding the individual decision making towards health insurance is imperative. This paper analyses the factors that affect the individual demand for private health insurance in Malaysia.

## Materials and methods

Data was extracted from the National Health and Morbidity Survey (NHMS) III. The NHMS III data was collected in year 2006 via self-administered questionnaire and interview. The analysis used dichotomous dependant variable which is a discrete choice of two options representing either the respondents have health insurance or not. As the dependant variable is discrete, a nonlinear probability model is employed in this study. The influences of predictive variables that determine the dependent variables were analyzed using logistic regression. The unit of analysis is the individual who is eligible to purchase health insurance. The independent variables include individuals’ socio-demographics characteristics and the imputed price of the health insurance product. The data set with 14,223 cases were randomly split into sub-samples, of which 7,069 cases were used to fit the model. The cases were then analyzed separately for salaried and non-salaried individuals, due to multicollinearity problem.

## Results

For the salaried individuals, it was found that income level, age, gender, race-religion, education level, job sector and risk attitude affected the decision to purchase while for the non-salaried individuals, the factors that affected the decision to purchase health insurance were race-religion, education level, marital status and out-of-pocket (OOP) health expenditures. The effect of price on the likelihood of purchase was found to be significant for the salaried individuals, but not for the non-salaried individuals.

## Conclusions

This study has identified the factors influencing individuals to purchase private health insurance. A national health insurance programme requiring individual premium contribution, and any intervention programme meant to increase individual participation, is likely to be more successful.

